# Patients and general practitioners assessment of the main outcomes employed in the acute and preventive treatment of migraine: a cross sectional study

**DOI:** 10.1186/s12883-021-02220-w

**Published:** 2021-07-15

**Authors:** Javier Trigo-López, Ángel Luis Guerrero-Peral, Álvaro Sierra, Enrique Martínez-Pías, María Gutiérrez-Sánchez, Elizabeth Huzzey, David García-Azorín

**Affiliations:** 1 Headache Unit, Neurology Department, Hospital Clínico Universitario de Valladolid, Avenida Ramón y Cajal 3, 47005 Valladolid, Spain; 2 Institute for Biomedical Research of Salamanca (IBSAL), Salamanca, Spain; 3 Department of Medicine, University of Valladolid, Valladolid, Spain; 4 OPHM, London, UK; 5 University of Copenhagen, Copenhagen, Denmark

**Keywords:** Migraine, Headache disorders, Patient outcome assessment, Treatment outcome, Patient reported outcome measures

## Abstract

**Background:**

We aim to describe and compare patients and general practitioners’ opinions about the different variables related to acute and preventive treatment for migraine.

**Patients and methods:**

An observational descriptive study was performed. Patients with episodic migraine and general practitioners, from our healthcare area, were invited to answer a survey about the different variables related to migraine treatment. They were asked for their opinions on the different variables, and to consider the desired efficacy in percentage terms and the desired action times of treatment.

**Results:**

Fifty-five patients and fifty-five general practitioners were selected. Effectiveness was considered the most important variable for symptomatic and preventive treatment. Cost was considered the least important variable. Patients desired percentage of efficacy was 84.0% (±16.7%) for symptomatic treatment and 79.9% (±17.1%) for preventive treatment. General practitioners desired percentage of efficacy was 75.0% (±14.0) for symptomatic treatment and 70.4% (±14.3) for preventive treatment. For symptomatic treatment the desired action time for pain cessation was selected as 27.5 min (±13.8) for patients and 24.0 min (±18.3) for GPs. For preventive treatment the desired action time for effect was 7.1 days (±4.5) for patients and 13.9 days (±8.9) for general practitioners.

**Conclusion:**

The most important endpoints were, for acute: effectiveness, a short action time and a persistent effect. For prophylactic: effectiveness, sustained effect and tolerability. Both patients and general practitioners agreed on the most and least preferred endpoints. Desired percentage of efficacy was above 75% for both symptomatic and preventive treatment; and the desired action time was below 30 min for acute treatment and 2 weeks for preventive treatment.

## Introduction

Migraine is the most disabling primary headache globally [1]. Despite being less prevalent than tension-type headache, it represents the second cause of years lived with disability in the world [1]. For its management we may act to abort migraine attacks with symptomatic treatments or, in some cases, trying to reduce monthly frequency of episodes through preventive treatments [2]. Fortunately, novel drugs have been developed and at present, other possible candidates are being studied [3].

In the validation of acute and preventive medications, International Headache Society (IHS) recommends harmonizing and standardizing the employed endpoints through IHS guidelines. In the case of acute treatment, the recommended main endpoint is the percentage of subjects who become pain free at 2 h after treatment [4]. For preventive therapies, there are three suggested primary efficacy endpoints: a change in migraine days, a change in moderate to severe headache days, or the 50% responder rate [5].

The choice for these outcome measures is based on consensus among experts [4, 5]. However, success is a matter of perspective and the endpoints that have been selected may not be fully accepted by patients or general practitioners (GPs). Understanding patient preferences and expectations may increase adherence and the chance for successful migraine management, so the current tendency is to consider and include patient related outcome measurements in the drug development process [6]. According to the Atlas of Headache Disorders [7], the bottom of the pyramid of headache management is primary care. GPs manage up to 90% of migraine patients. Their opinion and preferences seem to be, therefore, highly relevant.

In this study, we aim to analyze which endpoints are considered as most important by patients and GPs, to compare patients and GPs preferences, and to study the optimal effectiveness and action time in symptomatic and preventive treatment from their perspective.

## Methods

Observational descriptive study performed in two samples, patients and GPs. The first group included a series of consecutive cases of migraine patients attending the headache outpatient clinic of a tertiary hospital. The second group was composed of GPs who were contacted via institutional mail.

### Inclusion/exclusion criteria

Patients were included if 1) they were 18 years old and over and 2) had definite episodic migraine diagnosis according to the International Classification of Headache Disorders, 3rd version (ICHD-3) [8]. They were excluded if 1) they had previously used more than two acute or preventive medications; 2) they had another coexistent headache disorder other than infrequent episodic tension-type headache; 3) they had medication overuse headache or 4) they had another painful condition or any severe illness.

The second group consisted of a group of GPs from our healthcare area that were contacted through their institutional mail. All the GPs from our healthcare area were invited. We included all the GPs that provided information but we excluded responses from participants with 1) episodic migraine according to ICHD-3 [7] with more than two episodes per month in the preceding 3 months, 2) another coexistent headache disorder other than infrequent episodic tension-type headache; 3) medication overuse headache or 4) any other painful condition or any severe illness.

### Intervention

We analyzed demographic variables such as sex and age. We gave participants a list of endpoints, including a clear definition in plain language in Spanish (Table 1). We asked them for their opinion about acute and preventive treatment in two different ways: first we asked them to rate each variable on a 0–10 scale (0-worst possible, 10-best possible). We compared scores from patients and GPs. We also analyzed the differences and correlations in patients’ and GPs opinions when comparing acute and preventive treatments.

**Table 1:** The analysed endpoints with its explanation to patients, translated to English

Variable	Definition:
Action time	Speed to start acting
Tolerability	Lack of adverse events
Compatibility	Lack of interactions
Cost	Affordable price
Effectiveness	High probability to be effective
Sustained	A: Lack of rebound in case of efficacy.
	P: Sustained effect in case of efficacy.
Maintained	Preservation of effect in successive uses
Effect on other symptoms	Such as nausea, vomits

Second, they were asked to select the three most important variables and the least important endpoint. We compared the proportion of patients that answered each variable and the similarities between the groups.

In the case of preventive treatment, we asked specifically what they considered most important, the reduction in headache days per month, mean intensity of headache, the use of acute medication or the avoidance of emergency department visits.

We specifically analysed action times: we asked about the desired time to pain cessation, in the case of acute treatment. For prophylactic treatment, we asked about the desired time for effect and how long the treatment should be used, in case of efficacy.

The patients’ study took place between April 24th and May 31st, 2018. The GPs survey was sent on May 1st, with a reminder at May 15th. Ethics Review Board approved the study (PI-18-1028). All participants agreed to participate and signed an informed consent form.

### Statistics

Qualitative data is presented as frequency and percentage. Quantitative variables are presented as mean and standard deviation. We used **χ**^2^-squared test in the contrast of qualitative variables, detailing the degrees of freedom (df). In the comparison of qualitative variables between groups we used Student t test and in the comparison of acute and preventive treatment within groups we employed paired Student t test. We used Levene test in order to test homogeneity of variance. In the correlation tests we employed Pearson test. We accepted a *p* value lower than 0.05 as significant, correcting for multiple comparisons with Bonferroni method. Statistical analysis was done using SPSS for Mac (v23.0).

“We also analyzed the differences and correlations in patients’ and GPs opinions when comparing acute and preventive treatments. We assessed whether responders that considered a specific variable as relevant for the acute treatment, also judged it relevant for the prophylactic treatment”

## Results

During the study period, 182 patients were screened and 55 fulfilled inclusion/exclusion criteria. They were invited to participate and all of them accepted. Amongst the GPs, 198 were invited to participate and 67 answered the survey. We excluded 12 general practitioners because of frequent tension-type headache or migraine with more than two episodes in the preceding 3 months. Demographical variables are summarized in Table 2.

**Table 2:** demographical variables in patients and general practitioners (GPs). Values represent percentage for sex and mean age with standard deviation

	Patients	GPs	*p*-value
Female sex	81.2%	76.4%	0.55
Age	35.07 (11.4)	52.7 (9.8)	< 0.001

### Treatment evaluation

**Fig. 1:**
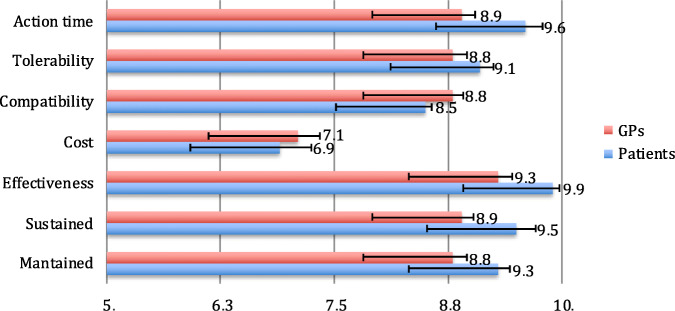
Migraine symptomatic treatment: mean score in a 0–10 scale (x axis) for the different endpoints (y axis) for General practitioners (GPs), in red, and patients, in blue

Figure 1 represents the scores of each variable related to acute treatment within the patients and GP groups. After adjusting for multiple comparisons, we found statistically significant differences in the score of speed (*p* < 0.001), effectiveness (*p* < 0.001), and persistency (*p* = 0.008).

**Fig. 2:**
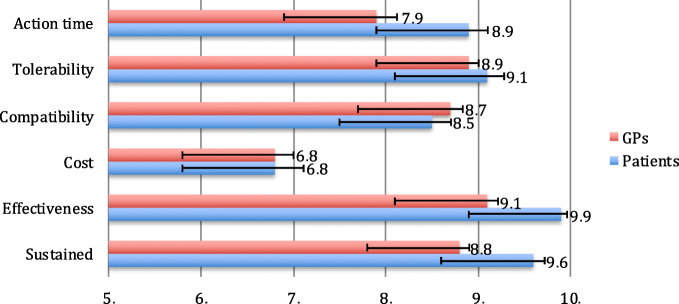
Migraine preventive treatment: mean score in a 0–10 scale (x axis) for the different endpoints (y axis) for General practitioners (GPs) in red, and patients, in blue

Concerning preventive treatment results are represented in Fig. 2. We found statistically significant differences in speed (*p* = 0.001), effectiveness (*p* < 0.001) and persistency (*p* < 0.001).

Tables 3 and 4 show the comparison between acute and preventive ratings within the GPs patient groups, and the correlation between those variables.

**Table 3:** Patients mean opinion about the different endpoints for acute and preventive treatment. Values represent the mean and standard deviation. Third column shows *p*-value of the statistical comparison between mean values for acute and preventive; correlation shows R-index of Pearson test, with the statistical value

	Patients				
	Acute	Preventive	*p*-value	Correlation	*p*-value
Action time	**9.6 (0.8)**	**8.9 (1.5)**	**0.004**	0.19	0.16
Tolerability	9.1 (1.4)	9.1 (1.3)	1.00	0.38	0.004
Compatibility	8.5 (1.8)	8.5 (1.5)	1.00	**0.66**	**< 0.001**
Cost	6.8 (2.4)	6.8 (2.3)	1.00	**0.89**	**< 0.001**
Effectiveness	9.9 (0.4)	9.9 (0.4)	1.00	−0.04	0.78
Sustained	9.5 (0.9)	9.6 (0.9)	0.13	**0.52**	**< 0.001**

**Table 4:** GPs’ mean opinion about the different endpoints for acute and preventive treatment. Values represent the mean and standard deviation. Third column shows *p*-value of the statistical comparison between mean values for acute and preventive; correlation shows R-index of Pearson test, with the statistical value

	GPs				
	Acute	Preventive	*p*-value	Correlation	*p*-value
Action time	**8.9 (1.0)**	**7.9 (1.6)**	**< 0.001**	**0.42**	**0.001**
Tolerability	8.8 (0.8)	8.9 (0.8)	0.24	**0.51**	**< 0.001**
Compatibility	8.7 (1.0)	8.7 (0.9)	0.56	**0.58**	**< 0.001**
Cost	7.1 (1.6)	6.8 (1.5)	0.06	**0.70**	**< 0.001**
Effectiveness	9.3 (0.7)	9.1 (0.8)	0.08	**0.63**	**< 0.001**
Sustained	8.9 (1.0)	8.8 (0.8)	0.41	**0.43**	**0.001**

When we compared the opinions for acute and preventive treatment.

In the comparison between acute and preventive treatment, in the patient’s group (Table 3) we found statistically significant differences concerning action time, considered more important in acute treatment than in preventive treatment (9.6 vs. 8.9, *p* < 0.004) and we found statistically significant positive correlation in the score of compatibility, cost and sustained effect (all *p* < 0.001).

In the case of GPs (Table 4), the comparison between acute and prophylactic treatment also showed that it was more important for acute than for preventive treatment (8.9 vs. 7.9, *p* < 0.001) and in the correlation analysis, cost and effectiveness had a strong correlation, compatibility, tolerability and sustained effect had a moderate correlation (all *p* = 0.001 or lower).

**Fig. 3:**
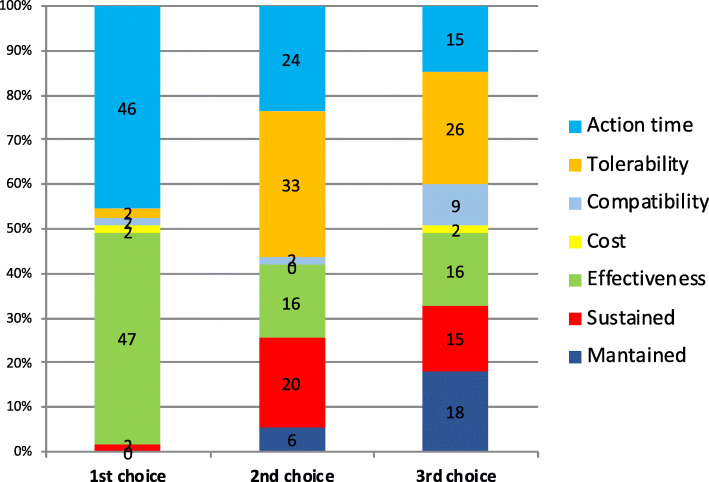
Patient’s’ top 3 preferred endpoints about symptomatic treatment (x axis). Percentage of participants that selected each of the different endpoints as first, second or third choice (y axis)

**Fig. 4:**
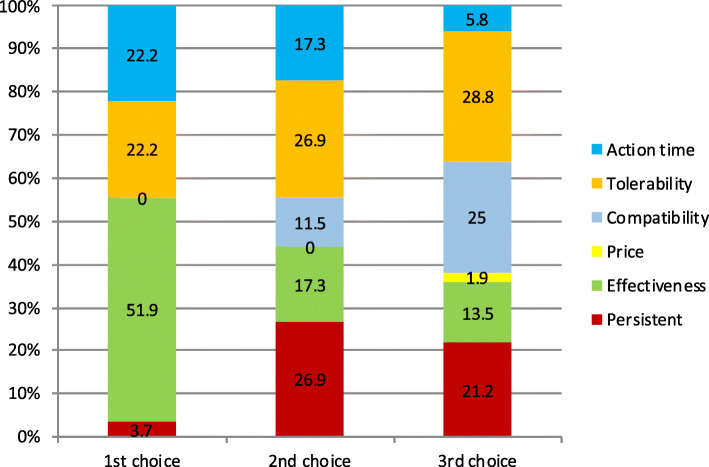
GP’s top 3 preferred endpoints about symptomatic treatment (x axis). Percentage of participants that selected each of the different endpoints as first, second or third choice (y axis)

Considering the three preferred endpoints, Fig. 3 shows patients’ choices for acute treatment and Fig. 4 the GPs’ choices. In the case of patients, we found statistically significant differences when comparing the first and second choice (**χ**^2^-squared test, 36 df, *p* < 0.001) but we did not reach statistical signification when comparing second and third choice (**χ**^2^-squared test, 30df, *p* = 0.24). We could not find differences in GPs choices between first and second choice (*p* = 0.11) or second and third (*p* = 0.01).

**Fig. 5:**
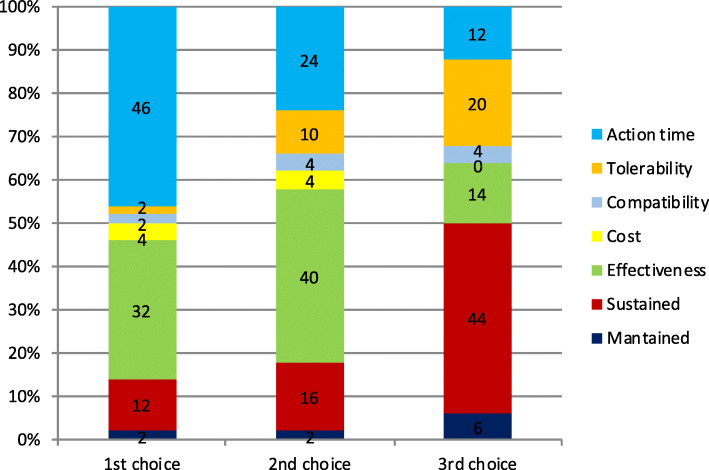
Patient’s top 3 preferred endpoints about preventive treatment (x axis). Percentage of participants that selected each of the different endpoints as first, second or third choice (y axis)

**Fig. 6:**
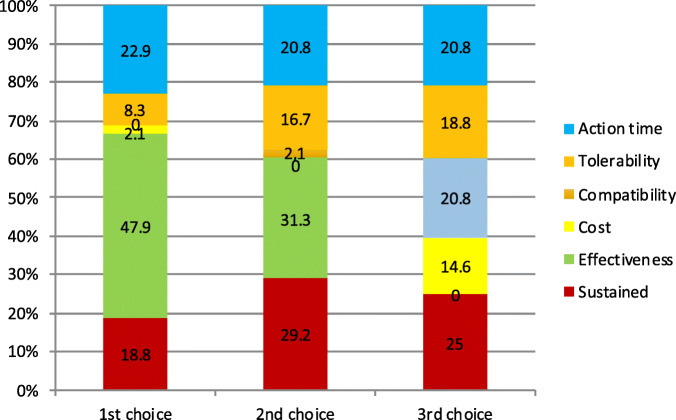
GP’s top 3 preferred endpoints about preventive treatment (x axis). Percentage of participants that selected each of the different endpoints as first, second or third choice (y axis)

With regards to preventive treatment, Fig. 5 shows patients’ selection and Fig. 6 GPs’ opinions. In the patients’ group, first and second choices were different (**χ**^2^-squared test, 16 df, *p* = 0.001), but second and third choices did not reach statistical signification after multiple comparison adjustment (**χ**^2^-squared test, 16 df, *p* = 0.032). In the GPs group, also first and second choices differed (**χ**^2^-squared test, 12 df, *p* = 0.002) and second and third (**χ**^2^-squared test, 24 df, *p* < 0.001).

**Fig. 7:**
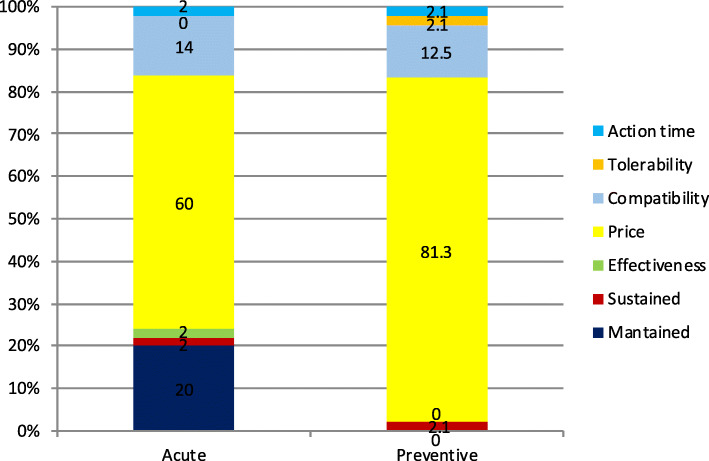
Least important variable for patients. x axis: on the left acute medication, on the right preventive medication. y axis: percentage of responders that selected each variable

**Fig. 8:**
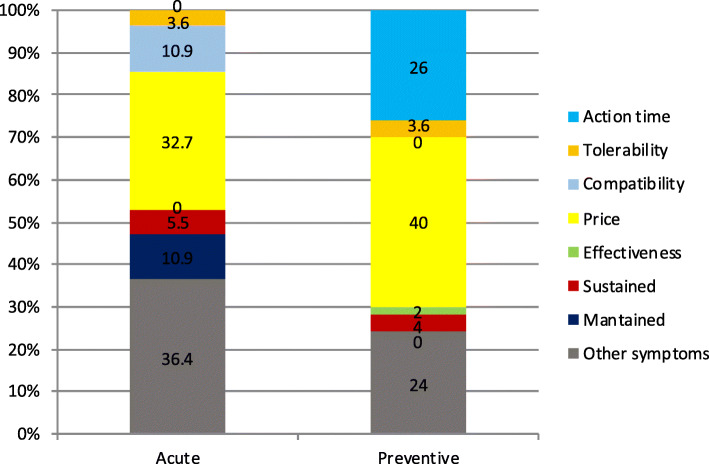
Least important variable for GPs. x axis: on the left acute medication, on the right preventive medication. y axis: percentage of responders that selected each variable

The least important variables for patients are showed in Fig. 7 and for GPs in Fig. 8. Differences in choices concerning acute treatment were statistically significant to those about preventive treatment in the patients’ group (**χ**^2^-squared test, 16 df, *p* < 0.001) and in the GPs group (**χ**^2^-squared test, 25 df, *p* < 0.001).

### Efficacy and time-related endpoints

In the case of acute treatment, the desired percentage of improvement to consider it as effective was set in 84.0% (16.7%) in the patients’ group, and 75.0% (14.0%) in the GPs group (Student t test, 108 df, *p* = 0.002). The desired time until pain cessation was selected as 27.5 min (13.8) for patients and 24.0 min (18.3) for GPs (*p* = 0.27).

For preventive treatment, the desired percentage of efficacy in order to consider the treatment as effective was selected as 79.9% (17.1%) for patients and 70.4% (14.3%) for GPs (Student t test, 100 df, *p* = 0.003). The desirable time to effect onset was 7.1 days (4.5) for patients and 13.9 days (8.9) for GPs (Student t test, 100 df, *p* < 0.001). The total desirable time of treatment was 3.0 (2.5) months for patients and 5.82 (2.6) months for GPs (Student t test, 83 df, *p* < 0.001).

## Discussion

In this study we aimed to evaluate the opinion of patients and GPs on the different variables related to symptomatic and preventive treatment for migraine. We performed a structured survey and participants gave their opinions about the most frequently employed variables.

The main findings of our study were that both patients and GPs considered effectiveness, a short action of time and persistence of the effect as the most important variables in symptomatic treatment. When considering preventive therapy effectiveness, sustained effect and tolerability were the highest rated variables. In contrast, cost was the lowest rated variable by both patients and GPs for both symptomatic and preventive treatment.

The level of patient’s satisfaction and their perception of treatment success usually depends on their previous expectations. There is generally a gap between what the patient expects from the treatment and the real result. The smaller this gap is, the higher level of satisfaction. In our study we found that almost all the scores were lower in the GPs group compared with patients, perhaps reflecting a better knowledge about the existing treatments. Patients must be properly informed on the expected effectiveness and tolerability of the different treatments. Furthermore, we should consider and integrate their opinions in research. A patient-reported outcome (PRO) is a health outcome directly reported by the patient who experienced it [9]. PRO are increasingly employed in clinical trials and clinical research, to help better understand a treatment’s or effectiveness [9].

### Effectiveness

Our study showed that effectiveness was the highest ranked variable. This finding concurs with previous studies [10–13] and is consistent with the IHS guidelines for clinical trials [4, 5], where effectiveness is recommended as the main endpoint for both symptomatic and preventive treatment. Guidelines not only improve quality of studies but also harmonize the different trials, making possible the combination of studies and meta-analysis. Nowadays, the approval of a new drug depends on the main endpoint compliance in pivotal clinical trials, reflecting how important the concept is.

According to the IHS guidelines definition, for acute prophylactic medications the primary efficacy endpoints should be the percentage of pain-free patients 2 h after the drug uptake [4]. In meta-analysis analyzing oral triptans, efficacy rate ranges from 18 to 50% [14]. In the case of prophylactic treatment, the suggested endpoints are the 50% responder rate or the decreased of headache days compared with baseline. Meta-analysis or oral preventive drugs show 50% responder rates that vary between 20 and 40% of the patients [15]. These percentages are lower than the desired percentage that we found in our study, above 75% both for symptomatic and preventive treatment. This reflects that researches should keep looking for novel targets and drugs with higher efficacy rates.

#### Action time

Action time was also considered among the most relevant variables. For symptomatic treatment, patients and GPs wanted action time within 30 min. This desired time is quite different to the 2 h recommended by IHS guidelines [4] and the 2 h which most triptans take to achieve a complete pain freedom [16]. In cases where a faster effect is needed, intranasal or subcutaneous formulations could be an interesting option [17, 18]. Regarding preventive treatment, patients considered it as the most important endpoint. IHS guidelines do not include it [5], so future versions might consider it as a secondary endpoint.

At present, action time of oral prophylactics tends to be slow, with the effectiveness usually assessed after a minimum of 4–8 weeks [19], far from the desired action time we found in patients, 7 days, or in GPs, 14 days. In contrast, newer treatments such as onabotulinumtoxinA (onabotA) or monoclonal antibodies (mABs) against Calcitonine Gene-Related Peptide (CGRP) have shown shorter efficacy onset, within hours or days [20, 21].

#### Sustained effect

Patients and GPs were also concerned about the enduring effect of treatments. In the case of acute medications, guidelines propose sustained pain-free response as a secondary endpoint [4]. Using triptans as an example, molecules with more prolonged half-life such as eletriptan, frovatriptan or rizatriptan [14] may be the treatment of choice in patients with a tendency to relapse. We differentiated a sustained effect from a maintained effect in order to discriminate it from tachyphylaxis, which is also shown as an important endpoint for patients.

With respect to preventive treatments, we analyzed if the persistence effect of the drug after treatment cessation was also important. This variable is usually among the least studied in trials before drugs commercialization and real-world data is also scarce. Some oral prophylactics may keep patients into a low frequency episodic migraine even after interrupted [22], whereas other drugs such as onabotA might need to be periodically administered to keep its effect [23]. Regarding mABs, data is scarce [24] and further research is needed to clarify this.

#### Tolerability

Although every physician considers tolerability when prescribing a medication, in our study we were surprised that patients and GP did not mention it as an important variable for symptomatic treatment. Fortunately, nowadays most symptomatic treatments are well tolerated if they are properly prescribed [25, 26]. In contrast, both patients and GPs considered tolerability a high rated variable for preventive treatment. Adverse events differ significantly depending on the therapeutic group, so patient prior medical history and comorbidities should be taken into account [27, 28].

#### Compatibility

Every time we prescribe a new drug, we should consider potential interactions with other drugs or conditions. In our study we found that it was not considered as a variable possibly reflecting the age of those taking the migraine drugs, the employed molecules [29] or because interaction with other drugs is rare.

#### Costs

Finally, cost was the lowest rated variable for both types of treatment. This result was expected. Spanish healthcare system provides public health services to the whole population and drugs are subsidized. Patients are requested to pay only a small proportion of the total cost or nothing at all. The results may be different if the study was performed in another country with a different healthcare system. Regardless, migraine is the second most disabling disease in the world [1] and the first for people in the productive years [1, 30, 31]. Cost analysis should consider not only the cost of the drug but other direct costs such as physician visits, emergency department visits or hospitalizations. There are also the indirect costs to productivity and employability of migraine sufferers [32]. Pharmacoeconomic studies consider cost as the most relevant parameter when analyzing the potential effect of a drug.

This study has some weaknesses, our groups were not balanced in terms of age, which could represent a selection bias. In addition, there could be a sampling bias affecting the external validity of the study as the sample were all within the same healthcare system and because chronic migraine patients have not been included. The response rate of General Practitioners was low. Despite it was similar to other studies published [33] and we controlled for prior history of headache, the responders could be selected based on a higher interest in headache disorders. Finally, the approach to patient opinions was made through closed questions, thereby not having the freedom to express their own opinions. We already mentioned that the study was done in a public health setting, which could limit generalizability of results.

As strengths, as far as we know, this is the first study in which GP opinions of the variables of migraine treatment has been assessed and then compared to patient opinions. All patients had a migraine diagnosis confirmed by experienced physicians and using ICHD.

Future studies should consider not only statistical signification but also clinical relevance. Evaluating patient opinion is crucial for the development of novel drugs able to satisfy the real demands and to increase patient’s satisfaction. Future studies may validate our results, including also patients with prior resistance to several preventive therapies and general practitioners with high-frequency episodic migraine or chronic migraine.

## Conclusion

1. The most important endpoints for acute treatment were effectiveness, short action time and persistence of the effect.
2. For prophylactic treatment, effectiveness, sustained effect and tolerability were the preferred endpoints.
3. Both patients and GPs agreed on the most and least preferred endpoints.
4. Desired percentage of efficacy was above 75% for both symptomatic and preventive treatment; and the desired action time was below 30 min for acute treatment and 2 weeks for preventive treatment.

### Clinical implications

1. Information to patients including realistic expectations of current treatments might improve patient satisfaction.
2. Action time is perceived as important for patients but not frequently considered in clinical trials.
3. Further research is needed to develop novel drugs with higher efficacy rates and shorter action time.

## Data Availability

The datasets used and/or analyzed during the current study are available from the corresponding author on reasonable request.

## Abbreviations

GPs: General practitioners
IHS: International Headache Society
ICHD-3: International Classification of Headache Disorders, 3rd version
PRO: A patient-reported outcome
onabotA: OnabotulinumtoxinA
mABs: Monoclonal antibodies
CGRP: Calcitonine Gene-Related Peptide
